# Physicochemical Characterization of Moroccan Honey Varieties from the Fez-Meknes Region and Their Antioxidant and Antibacterial Properties

**DOI:** 10.3390/metabo14070364

**Published:** 2024-06-27

**Authors:** Atika Ailli, Khalid Zibouh, Brahim Eddamsyry, Aziz Drioiche, Dounia Fetjah, Fatima Zahra Ayyad, Ramzi A. Mothana, Mohammed F. Hawwal, Mohamed Radi, Redouane Tarik, Abdelhakim Elomri, Aicha Mouradi, Touriya Zair

**Affiliations:** 1 Research Team of Chemistry of Bioactive Molecules and the Environment, Laboratory of Innovative Materials and Biotechnology of Natural Resources, Faculty of Sciences, Moulay Ismaïl University, B.P. 11201, Zitoune, Meknes 50070, Morocco; a.ailli@umi.ac.ma (A.A.); k.zibouh@edu.umi.ac.ma (K.Z.); b.eddamsyry@edu.umi.ac.ma (B.E.); d.fetjah@umi.ac.ma (D.F.); f.ayyad@edu.umi.ac.ma (F.Z.A.); m.radi@edu.umi.ac.ma (M.R.); re.tarik@edu.umi.ac.ma (R.T.); a.mouradi@umi.ac.ma (A.M.); 2 Department of Pharmacognosy, College of Pharmacy, King Saud University, Riyadh 11451, Saudi Arabia; rmothana@ksu.edu.sa (R.A.M.); mhawwal@ksu.edu.sa (M.F.H.); 3 INSA Rouen Normandy and CNRS, Laboratory of Organic, Bioorganic Chemistry, Reactivity and Analysis (COBRA-UMR 6014), Medical University of Rouen Normandy, 76000 Rouen, France; hakim.elomri@univ-rouen.fr

**Keywords:** honey, antimicrobial activity, polyphenols, flavonoids, DPPH, FRAP, phytochemical properties

## Abstract

Honey, with its varied and extensive characteristics, is a complex and diverse biological substance that has been used since ancient times. The aim of this study is to thoroughly characterize the physicochemical, phytochemical, and biological properties of four floral honey varieties from the Fez-Meknes region in Morocco, with the goal of promoting the valorization of Moroccan honey in skincare and cosmetic products. The analyses of their physicochemical characteristics encompass various parameters such as pH, acidity, density, water content, Brix index, conductivity, ash content, hydroxymethylfurfural (HMF) content, and color. The levels of polyphenols range from 22.1 ± 0.4 to 69.3 ± 0.17 mg GAE/100 g of honey, measured using the Folin–Ciocalteu method for polyphenol quantification. Additionally, the estimation of flavonoid quantities in 100 g of honey, conducted using the aluminum trichloride method, reveals values ranging from 3.6 ± 0.2 to 7.2 ± 0.6 mg QE. Furthermore, it is noteworthy that honey exhibits high levels of glucose and relatively low concentrations of proteins. The quantitative evaluation of antioxidant effects, carried out through the 2,2-diphenyl-1-picrylhydrazyl free-radical-scavenging method and the ferric-reducing antioxidant power (FRAP) method, highlights the strong antioxidant capacity of multifloral honey, characterized by low inhibitory concentration values (IC_50_ = 30.43 mg/mL and EC_50_ = 16.06 mg/mL). Moreover, all honey varieties demonstrate antibacterial and antifungal properties, with multifloral honey standing out for its particularly pronounced antimicrobial activity. The correlation analyses between phytochemical composition and antioxidant and antibacterial activities reveal an inverse relationship between polyphenols and IC_50_ (DPPH) and EC_50_ (FRAP) concentrations of honey. The correlation coefficients are established at R^2^ = −0.97 and R^2^ = −0.99, respectively. Additionally, a significant negative correlation is observed between polyphenols, flavonoids, and antifungal power (R^2^ = −0.95 and R^2^ = −0.96). In parallel, a marked positive correlation is highlighted between antifungal efficacy, DPPH antioxidant activity (R^2^ = 0.95), and FRAP (R^2^ = 0.92). These results underscore the crucial importance of phytochemical components in the beneficial properties of honey, meeting international quality standards. Consequently, honey could serve as a natural alternative to synthetic additives.

## 1. Introduction

Oxidative stress is defined as an imbalance between the production of oxidants and the activity of antioxidant systems, leading to structural and functional damage to essential biomolecules such as nucleic acids, lipids, and proteins [1,2]. To tackle this oxidative stress without depending on synthetic antioxidants, scientists have directed their attention towards the examination of substances that are naturally abundant in phenolic compounds, which are recognized for their strong antioxidant capabilities [3,4].

Honey, a natural sweet and viscous substance produced by bees (*Apis mellifera*) from flower nectar and honeydew, constitutes an important source of antioxidants, and is believed to have therapeutic qualities [5]. Honey has been used for centuries and is still highly valued in many fields. According to the literature, flavonoids, vitamins, phenolic acids, minerals, and certain enzymes are the primary sources of its antioxidant activity [6].

Other studies have shown that the antioxidant activity of honey primarily stems from its phenolic compound content [7], with this composition varying greatly depending on the floral source and botanical origin [8]. Honey’s high antioxidant activity, which includes phenolic acids and flavonoids, makes it an intriguing therapy option for herpes outbreaks [9]. These compounds play an essential role in honey’s antioxidant capacity, thus protecting cells against the damaging effects of free radicals. This antioxidant activity contributes to honey’s beneficial effects on health, particularly in the treatment of herpes-related outbreaks. Thus, the phenolic chemicals and flavonoids present in honey’s composition play a major role in its therapeutic qualities.

Beyond its well-documented antioxidant properties, honey has also been the subject of extensive research regarding its antimicrobial and antiviral capabilities [10]. Rigorous in vivo and in vitro investigations have elucidated honey’s remarkable ability to stimulate the proliferation of beneficial bacterial strains, such as lactobacilli [9]. These probiotic effects are particularly noteworthy, as honey has demonstrated the capacity to selectively promote the growth of commensal microorganisms while concurrently exerting inhibitory influences on a wide array of pathogenic bacteria, including those resistant to conventional antibiotic treatments [11]. The antimicrobial potency of honey is attributable to a multifaceted mechanism of action involving the synergistic contributions of its naturally occurring bioactive constituents. These include, but are not limited to, the enzymatic generation of hydrogen peroxide, the presence of proteinaceous antibacterial compounds, and the osmotic stress imposed by honey’s elevated sugar content [12]. Intriguingly, honey has also exhibited pronounced antiviral activity in vitro, displaying inhibitory effects against viral agents such as rubella, leishmaniasis, and echinococcosis [9]. The antiviral efficacy of honey is of particular clinical relevance, as it has been shown to be an effective topical treatment for both labial and genital manifestations of the herpes simplex virus [13,14,15,16]. This therapeutic potential is likely attributable to honey’s multifaceted bioactivities, including its capacity to modulate host immune responses, disrupt viral replication cycles, and mitigate the oxidative stress associated with viral infections. Collectively, these antimicrobial and antiviral properties underscore honey’s considerable promise as a natural, broad-spectrum agent for the management of diverse infectious diseases.

The biological activities of honey are closely related to its complex and diverse chemical composition, comprising carbohydrates, organic acids, proteins, minerals, vitamins, lipids, and trace elements [17,18,19,20]. Potassium is the most abundant mineral, representing more than 30% of the total mineral content [21]. Carbohydrates, particularly fructose and glucose, constitute the main components, accounting for approximately 95% of the dry matter [20]. Though present in low concentrations, vitamins, especially those of the B group and vitamin C, are better preserved due to honey’s low pH [22].

In order to ensure the quality of honey and fully benefit from its antioxidant and antimicrobial virtues, it is essential to determine its physicochemical properties, which provide information on its degree of degradation during storage [23]. A high water content, for example, could promote the growth of yeasts and microorganisms that ferment sugars, thus modifying the flavor of honey [24]. Electrical conductivity and ash content indicate the presence of organic acids, major minerals, and trace elements, factors that influence microbial growth [25]. The physicochemical properties of honey are also dependent on the types of pollen and nectar taken from its plant sources, affecting its nutritional value [26].

This study aims to determine the physicochemical properties of four honey samples of different botanical origin, as well as to evaluate their antioxidant and antimicrobial capacities in relation to their phytochemical composition.

## 2. Materials and Methods

### 2.1. Sampling

The investigation focused on four unique honey varieties, sourced in 2021 from diverse locations across the Fez-Meknes region. Further details can be found in Table 1. Samples of this precious nectar were collected from beekeepers at production sites located in four distinct areas of the Fez-Meknes region in the Kingdom of Morocco. These honey specimens originated from hives belonging to cultivators who direct their bee colonies towards fields characterized by a flora known to yield honeys with singular organoleptic properties, depending on the predominantly foraged plant species. The beekeepers attested to the botanical origin of the honeys based on their sensory characteristics (color, aroma, flavor, texture, etc.). Prior to any analysis or utilization, the samples were stored at a temperature of 4 °C in hermetically sealed containers.

### 2.2. Sampling Physicochemical Analysis of Honey

#### 2.2.1. Determining the Hydrogen Potential of Honey

The pH of honey is influenced by the quantity of ionizable acids and minerals it contains. One of the main factors influencing the spontaneous breakdown of honey during natural aging or heating is the pH at the time of potting, which in turn determines the pH [27].

The hydrogen potential of a solution of 10 g honey diluted in 75 mL distilled water was determined using a pH meter that had previously been calibrated using buffer solutions (pH = 4 and pH = 7). The pH reading appears on the pH meter’s screen once it has stabilized.

#### 2.2.2. Determination of Honey-Free Acidity

Free acidity is obtained by neutralizing 20 mL of a 10% honey solution with NaOH (0.1 N) in the presence of bromothymol blue (BBT), an end-of-equivalency indicator. The following relationship (1) provides the free acidity levels, which are expressed in mEq/Kg.

(1) $$Free\;Acidity=\frac{1000.Veq.N}{m}\;(\mathrm{meq}/\mathrm{Kg})$$

Veq: Equivalent volume of NaOH in (L).

N: NaOH solution normality (eq/L).

m: Test sample of honey solution in kg.

#### 2.2.3. Determination of the Water Content and Brix Level of the Honey Studied

The Brix level is determined by placing a drop of honey on a refractometer prism that has already been calibrated with distilled water. Readings are displayed on the screen at 20 °C. This measurement indicates the grams of sugar contained in 100 g of honey. Then, other components with low amounts are estimated by deducting the proportion of water.

#### 2.2.4. Determination of Honey Density

A pycnometer is a tool used to measure density. Density is calculated by dividing the density of honey at the same conditions by the density of distilled water.

The following Formula (2) is used to determine the density (D) of honey.

(2) $$D=\frac{Pycnometer\;mass\;containing\;honey-Empty\;pycnometer\;mass}{Pycnometer\;mass\;containing\;distilled\;water-Empty\;pycnometer\;mass}\;(g/{\mathrm{cm}}^{3})$$

#### 2.2.5. Determining the Ash Content of Honey

Anklam (1998) [28] states that ash content is a quality attribute that depends on the honey’s botanical origin; changes in this criteria are associated with mineral component concentrations [29]. We utilized pre-weighed capsules containing 3 g of honey. The honey is heated to 500 °C for three hours, or until the organic material has completely burnt. After weighing the capsule containing the honey, the ash content is calculated using Formula (3):

(3) $$Ash\;content=\frac{\left(M_{2}-M_{1}\right)}{M_{0}}\times 100\;(\%)$$

M_0_: Honey mass in g.

M_1_: Mass of empty capsule in g.

M_2_: Mass of capsule with ash in g.

#### 2.2.6. Determination of Electrical Conductivity

A conductivity meter is used to determine each honey sample’s electrical conductivity. The electrical resistance at 20 °C serves as the method’s foundation. A small amount of distilled water is used to dissolve a 20 g lump of honey. A 100 mL flask is filled with the solution, which is then measured and adjusted. The honey solution is poured into a 40 mL thermostated bath at 20 °C. Once the temperature has stabilized, the conductivity, which is measured directly on the device and expressed in milliSiemens per centimeter, is read. An inoLab^TM^ Cond 7310 conductivity meter (WTW, inoLab Oxi 7310, Berlin, Germany) was used. 

#### 2.2.7. Determination of Hydroxymethylfurfural (HMF) Content

An amount of 5 g of honey should dissolve in 25 mL of distilled water in a beaker. Subsequently, the blend is transferred into a 50 mL flask. To make the solution more transparent, add 0.5 mL of Carrez solutions I (15 g of potassium hexacyanoferrate in 100 mL of distilled water) and II (30 g of zinc acetate in 100 mL of distilled water) before adding more distilled water to the mixture. To remove the froth that forms, add a drop of ethanol. As a reference solution, we prepared a solution of sodium meta-bisulfite diluted in 100 mL of distilled water (0.2 g/mL) [30]. 

In the first test tube, 5 mL of the filtered solution and 5 mL of distilled water are mixed. The second tube is filled with 5 mL each of the same solution and the sodium metabisulfite solution. The absorbances of the reference solution and the sample solution are compared at 284 and 336 nm.

The following Formula (4) is used to compute the HMF content:

(4) $$HMF=\frac{\left(A_{284}-A_{336}\right)\times 149.7\times 5\times D}{M}\;(\mathrm{mg}/\mathrm{kg})$$

A_284_: Absorbance at 284 nm. 

A_336_: Absorbance at 336 nm.

M: Honey sample mass in g.

D: Dilution factor (if dilution is necessary).

#### 2.2.8. Determination of Honey Color

Color is one useful characteristic for classifying single-flower honey. Honey’s hue can be determined by its botanical origin: the higher the mineral content, the darker the honey. It is assessed using spectrophotometry. Honey’s color intensity is measured using a PFUND index scale. 

An amount of 1 g of honey is dissolved in 4 mL of distilled water. After homogenization, the spectrophotometer measures the optical density of the honey solution at 450 nm.

#### 2.2.9. Determination of Glucose Content

The amount of glucose in the honey samples was measured using a commercial kit based on the glucose oxidase technique (Abbott Diagnostics, Lake Forest, IL, USA) [31]. After mixing 500 µL of reagent with 5 µL of either the sample or the standard, the mixture was incubated at 37 °C for 10 min. The absorbance of standards or samples was measured at 520 nm against a blank for 60 min using a multi-mode reader.

#### 2.2.10. Determination of Protein Content

Protein analysis was performed according to Bradford (1976), using the Bradford technique [32]. Accordingly, 4 mL of Coomassie brilliant blue (BBC) was combined with a 100 µL volume of honey solution (1 g/mL distilled water). The absorbance was measured against a blank (100 µL distilled water with 4 mL BBC) after stirring for 5 min. Using a calibration curve created under identical experimental circumstances using a 1 mg/mL solution of bovine serum albumin (BSA), the quantity of protein was calculated at 595 nm.

### 2.3. Phytochemical Study of Honeys

#### 2.3.1. Determination of Polyphenol Content

Singleton’s [33] Folin–Ciocalteu technique was utilized to ascertain the concentration of phenolic content in the honey solutions. By using this technique, phosphotungstic (H_3_PW_12_O_40_) and phosphomolybdic (H_3_PMo_12_O_40_) acids are reduced to a mixture of blue oxides of molybdenum (Mo_8_O_3_) and tungsten (W_8_O_23_). After adding 5 mL of a diluted honey solution to 5% (*w*/*v*) distilled water, 1.5 mL of a diluted Folin–Ciocalteu reagent in 10% distilled water, and 1.5 mL of 7% (*w*/*v*) Na_2_CO_3_, fill a 50 mL volumetric flask halfway with distilled water. After an hour of room-temperature incubation and light shielding, a reading is taken using a spectrophotometer (UV mini-1240, Shimadzu Corp., Kyoto, Japan) at 760 nm against a blank. Under the same operating conditions, the calibration curve is run simultaneously with gallic acid serving as a positive control. The findings are shown as gallic acid equivalents in mg per 100 g of honey.

#### 2.3.2. Determination of Flavonoid Content

The colorimetric approach of Djeridane and Hung [34,35] was utilized to evaluate the flavonoid concentration. The method involves the use of aluminum chloride, which produces a yellow complex with phenolic chemicals.

Indeed, 1 mL of AlCl_3_ (2%) in methanol is combined with 1 mL of honey solution (5 g/100 mL methanol). A spectrophotometer (UV mini-1240) set to 433 nm is used to take the reading against a blank following a half-hour dark incubation at room temperature. Quercetin is used as a positive control and the calibration curve is performed concurrently under the same operating conditions. The results are given in mg (mg QE/100 g honey) of quercetin equivalent per 100 g of honey.

### 2.4. Biological Study of Honeys

#### 2.4.1. DPPH* Free-Radical-Scavenging Effect

The ability of an antioxidant to stabilize the DPPH* radical (violet) into DPPH-H (yellow) is the basis for evaluating free-radical-scavenging activity [36]. A spectrophotometer was used to measure the absorbance at 515 nm after 30 min at room temperature, protection from light, and incubation in the presence of 2.8 mL of the ethanolic solution of DPPH* (2.4 mg DPPH*/100 mL absolute ethanol (purity > 99.8%)) or 200 µL of various sample concentrations or the standard antioxidant (ascorbic acid). As a negative control, DPPH* in an ethanolic solution without honey was used. The following Formula (5) was used to compute the percentage of antioxidant activity, also known as percentage inhibition:

(5) $$AA=\frac{A_{control}-A_{sample}}{A_{control}}\times 100(\%)$$

AA%: Percentage of antioxidant activity (percentage of inhibition).

A_control_: Absorbance of the solution containing only the DPPH* radical solution.

A_sample_: Absorbance of the test sample solution in the presence of DPPH*.

#### 2.4.2. Evaluation of Reducing Power (FRAP)

The method by Končić [37] is used to evaluate the capacity of honey solutions to achieve ferrous iron (Fe^2+^), which is present in the potassium ferricyanide complex, from ferric iron (Fe^3+^). 

Initially, 0.5 mL of various concentrations of honey solutions, 2.5 mL of a 0.2 M phosphate-buffered solution (Ph = 6.6), and 2.5 mL of a 1% potassium ferricyanide K_3_Fe(CN)_6_ solution are combined in test tubes. To halt the process, 2.5 mL of 10% trichloroacetic acid is added after the mixture has been incubated for 20 min at 50 °C in a water bath. Then, 2.5 mL of the supernatant from each concentration is combined with 0.5 mL of an aqueous solution containing 0.1% FeCl_3_ and 2.5 mL of distilled water. A spectrophotometer was used to assess absorbance at 700 nm in comparison to a blank. Ascorbic acid was used as the positive control. A calibration curve was created using six different doses of ascorbic acid, ranging from 10 to 200 µg/mL. According to Balouiri [38], there is a direct correlation between the reducing power of honey solutions and their absorbance.

#### 2.4.3. Determination of Antimicrobial Activity

To evaluate the antibacterial activity of the honey under investigation, we employed ten microorganisms, as detailed in Table 2, comprising five bacterial and five fungal strains, utilizing the micro-dilution technique [39]. Pathogenic microorganisms often associated with diseases in Morocco were taken from two hospital settings: The Hospital Provincial Mohamed V-Meknes and the mycotheque of the Parasitology–Mycology Laboratory of the Ibn Sina Hospital Center–Rabat.

The BD Phoenix (Becton, Dickinson and Company, Franklin Lakes, NJ, USA) and VITEK2 (bioMérieux, Marcy-l’Étoile, France) automated systems were used to perform the antibiogram of the selected strains against the antibiotics (Gentamycin; Amoxicillin–Clavulanate; Vancomycin and Trimethoprim-Sulfamethoxazole). The clinical interpretation of antimicrobial susceptibility was conducted according to the current EUCAST guidelines [40,41]. This antibiogram process with these automated systems involves evaluating bacterial growth in the presence of the antibiotic at different concentrations.

Using a stock honey solution with a concentration of 200 mg/mL, a series of dilutions (1/2, 1/4, 1/8, 1/16, 1/32, 1/64) were prepared. In each well of a sterile microtiter plate, 100 µL of Sabouraud broth for fungi and Mueller–Hinton broth for bacteria were added. Next, we diluted the stock solution from highest to lowest concentration, adding 100 µL to the first well. Subsequently, 10 µL of 10^6^ CFU/mL and 10^4^ CFU/mL of inoculum for the bacteria and fungi were introduced into each well. We added 10 µL of resazurin, an indication of microbial growth, at a concentration of 5 mg/mL to each well of the microplate after it had been incubated for 24 to 48 h at 37 °C. A second incubation at 37 °C for two hours reveals microbial development when the color changes from violet to pink.

The lowest concentration of honey that completely inhibits the development of microorganisms is known as the minimum inhibitory concentration (MIC) (i.e., prevents the color change of the resazurin). Taking 10 µL from each well that shows no visible growth yields the minimum fungicidal concentration (MFC) and minimum bactericidal concentration (MBC). After that, these samples are put onto plates that have either fungi or bacteria infected with Mueller–Hinton agar or Sabouraud. Following a 24 h incubation period at 37 °C, the lowest concentrations of MBC and MFC were shown to result in a 99.99% decrease in CFU/mL compared to the control. Antimicrobial potency is determined by calculating the ratio of MBC/MIC or MFC/MIC. If the ratio is less than 4, the honey’s influence is bactericidal/fungicidal; if the ratio is more than 4, the sample has a bacteriostatic/fungistatic effect. The 11th and 12th wells in each series are regarded as growth and sterility controls, respectively. The test is performed twice on each sample.

### 2.5. Statistical Study

The results, together with their corresponding standard deviations, represent the means of three separate trials. The data were analyzed utilizing the GraphPad Prism 9 software (version 9.5.1) developed by GraphPad Software Inc. (San Diego, CA, USA). One-way analysis of variance (ANOVA) was used to assess the significant differences among several groups. Significance was attributed to differences with a *p*-value less than 0.05. In order to examine the connections and associations between antioxidant activity, antibacterial activity, and the levels of polyphenols and flavonoids in four different types of honey, we utilized the R software (version 4.1.3) to create a heatmap and calculate the Pearson correlation coefficients.

## 3. Results and Discussion

Four samples were collected from different sites in the Fez-Meknes area to contribute to improving the value of Moroccan honey. After determining the samples’ physicochemical characteristics, the honeys were examined for their protein, polyphenol, and flavonoid contents. They were also evaluated for their antioxidant and antimicrobial qualities. Lastly, an analysis was conducted to determine the relationship between the honeys’ composition in terms of polyphenols and flavonoids and their antioxidant and antimicrobial properties.

### 3.1. Physicochemical Analyses

Table 3 below displays the findings for the honeys that were examined in terms of pH, free acidity, Brix level, water content, density, ash content, conductivity, hydroxymethylfurfural content, and color.

#### 3.1.1. Determination of Hydrogen Potential

Given that the honey samples’ pH values ranged from 2.91 ± 0.01 to 3.51 ± 0.01, it may be assumed that the honey under study was made from nectar. These findings are consistent with the Malaysian honey range of 2.51 to 3.26 [39]. 

The appropriate acidity of the pH prevents or just slows the development of several bacterial species [43]. Variations in pH can be explained by some variables, the most important of which are the floral origin, the extraction method, and the storage conditions. 

#### 3.1.2. Determining Free Acidity

Acidity is an important criterion for assessing the quality of honey since it rises with fermentation and offers insightful information about the honey’s condition. The honey that was the subject of the inquiry had free acid concentrations that ranged from 8.0 ± 0.1 to 13.0 ± 0.1 meq/kg, below the 50 meq/kg maximum permitted by the Food Codex Alimentarius of 2001. Therefore, we may conclude that none of the four honeys had accidentally fermented. These numbers are a great deal lower than those for honey from the Ivory Coast. The acidity levels of honey from the Yamoussoukro region vary from 42.6 mEq/Kg to 43.53 mEq/Kg, according to Iritié [44]. Conversely, Kouamé [45] found that Agboville (Cechi sub-prefecture) honey had acidity levels ranging from 15.00 mEq/Kg to 25.00 mEq/Kg. Various parameters such as flower origin, harvest season, extraction method, and storage conditions might impact the free acidity of honey varieties [46].

#### 3.1.3. Determining the Brix Value

The range of Brix was 80.2 ± 0.38 to 82.1 ± 0.41, with an opposite relationship to water content. These findings are similar to those made by Kouamé and vary from 76.50 ± 0.10% to 81.40 ± 0.01% for honey from the Ivory Coast [45].

#### 3.1.4. Determination of Water Content

While multi-flower honey had a relatively high water content (19.8 ± 0.38%), carob honey had a low water content (17.9 ± 0.42). Both jujube and rosemary honey had the same amount of moisture. The water levels of all four samples met the 20% barrier that the Food Codex Alimentarius of 2001 set as the gold standard for honey maturity and quality. Weather and the time of year that crops are harvested could cause variations. Typically, excessive moisture content (over 20%) may cause honey to ferment and lose its flavor and quality [47]. According to the Food Codex Alimentarius of 2001, this becomes uncommon when the honey concentration is less than 17%. As a result, carob honey will have a longer shelf life than other honey.

#### 3.1.5. Determination of Honey Density

The densities of the honey samples ranged from 1.412 ± 0.020 to 1.437 ± 0.050 g/cm^3^. Multi-flower honey had the highest density, with carob honey having the highest density at 1.437 ± 0.001 g/cm^3^, followed by jujube honey at 1.421 ± 0.002 g/cm^3^ and rosemary honey at 1.426 ± 0.001 g/cm^3^. For honey from Béjaïa (Algeria), Ouchemoukh found density values ranging from 1.401 to 1.451 g/cm^3^ [48]. These variations can be partially attributed to water content; also, honey harvested too soon or from damp circumstances contains too much water [49], which reduces its density.

#### 3.1.6. Determination of Ash Content

The ash contents of the samples analyzed ranged from 0.29 ± 0.01% to 0.6 ± 0.01%. Consequently, they satisfy the requirement of ash content less than or equal to 0.6% for nectar honey set by Codex Alimentarius. Due to the presence of more foreign substances and mineral particles, jujube honey had the highest proportion of ash. The different ash content levels can be explained by the physiology of the foraged plants, differences in soil types in the collection locations, and the unique climate in different areas.

#### 3.1.7. Determining Electrical Conductivity

Electrical conductivity is a significant method for determining the botanical origin of honey. In the current examination, the electrical conductivity values ranged from 0.199 ± 0.01 mS/cm to 0.473 ± 0.01 mS/cm. Jujube honey has a greater conductivity. The values reported by Iritié (0.596–0.707 mS/cm) are much higher than ours [44], while the values obtained by Kouamé [45] are extremely low (53.80 ± 0.17–129.47 ± 0.38 mS/cm). These readings are less than the upper limit (0.8 mS/cm) that European guidelines prescribe. These variations may be caused by the kind of plants harvested for complex honey, the phytogeographical context, or external variables [50].

#### 3.1.8. Determination of Hydroxymethylfurfural (HMF) Content

The ideal settings for preserving honey state that the HMF level is a great indicator of the honey’s condition since it rises with age and becomes more acidic with heat [51]. The exceptionally acidic pH of the honey may be the reason for the rosemary honey’s HMF levels, which were 48.65 ± 0.51 mg/Kg, slightly above the 40 mg/Kg Codex Alimentarius regulations. This requirement was met by the HMF levels of the following honeys: multi-flower (37.5 ± 0.24 mg/Kg), jujube (35.5 ± 0.26 mg/Kg), and carob (30.34 ± 0.44 mg/Kg).

#### 3.1.9. Color Determination

The honey under examination varies in color from light yellow to dark brown. The PFUND Index measurements for rosemary honey range from 313 ± 3 mm Pfund to 1202 ± 8 mm Pfund. Conversely, the findings for jujube and carob honeys are 622 ± 6 mm Pfund and 558 ± 4 mm Pfund, respectively. This variety is connected to the honey’s phenolic, pollen, and mineral contents. In fact, the more minerals and phenolic chemicals a honey has, the darker it gets. The process used in creating the honey and the conditions under which it is stored (temperature, time) are two further variables that might affect its hue.

### 3.2. Phytochemical Analysis

#### 3.2.1. Determination of Polyphenol and Flavonoid Content

**Figure 1:**
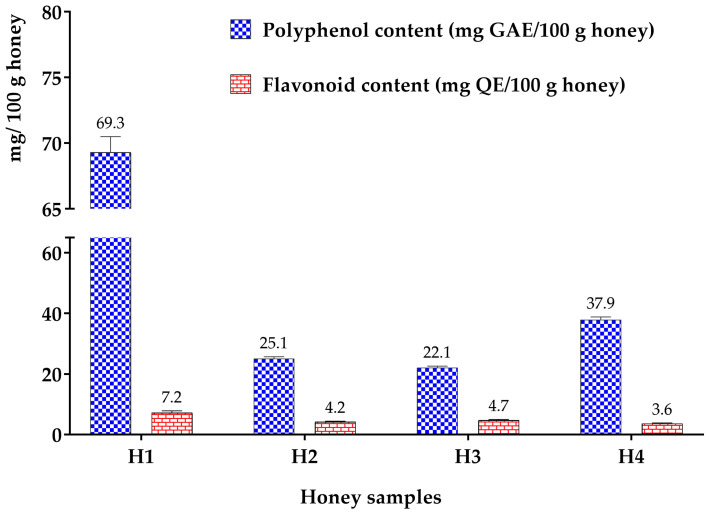
Polyphenol and flavonoid contents of multi-flower honey, jujube honey, carob honey, and rosemary honey.

The colorimetric approach is employed to ascertain the total contents of polyphenols and flavonoids in the tested honey samples. Figure 1 illustrates these concentrations for polyphenols in gallic acid equivalent and flavonoids in quercetin equivalent.

The amount of polyphenols in honey ranges from 22.1 ± 0.4 to 69.3 ± 0.17 mg GAE/100 g. The concentration was highest in the honey containing many flowers and lowest in the honey including carob. These results are significantly lower the range of 75.13 to 246.21 mg GAE/100 g for Italian honey published by Al-Mamary [52]. In comparison, Perna [53] reported that their research on honey from southern Italy yielded an average of 12.06 mg GAE/100 g. Bouyahya [54] showed that the polyphenol concentrations in their investigation of Ouazzane (Morocco) honeys ranged from 75.14 ± 0.78 to 124.60 ± 1.12 mg GAE/g honey. According to Bakchiche [46], variations in polyphenol content can be ascribed to the honey’s botanical origin. Multi-flower honey has the highest flavonoid concentration (7.2 ± 0.6 mg QE/100 g honey), whereas rosemary honey has the lowest (3.6 ± 0.2 mg QE/100 g honey). Values ranging from 6.61 to 28.05 mg QE/100 g honey were reported in Saric’s study [55] on Croatian honey, which is above these limits. However, according to Kadri [56], the range of flavonoid concentration in Brazilian honey is 3.30 to 3.63 mg QE/100 g of honey. On the other hand, readings ranged from 12.7 to 109.4 mg QE/100 g of honey, according to Habib [57]. Since most metabolites are created in response to stress, this variation may be explained by the type and severity of stress that the plant is experiencing, as well as the botanical origin of the nectar that the bees are collecting [58].

#### 3.2.2. Determination of Glucose and Protein Content

**Figure 2:**
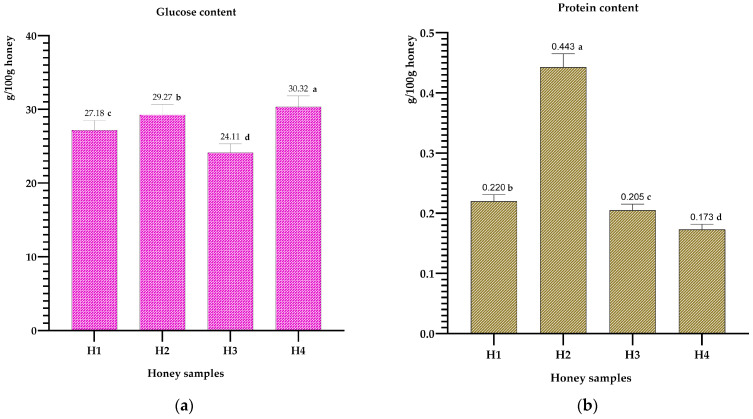
Glucose (**a**) and protein (**b**) content of multi-flower honey, jujube honey, carob honey, and rosemary honey. The results with different letters are significantly different from each other (*p* < 0.05).

The concentrations of glucose and proteins in honey are presented in Figure 2 below. This illustration highlights the protein contents of the different tested honeys, ranging from 0.173 g/100 g (rosemary honey) to 0.443 g/100 g (jujube honey). However, White and Gonnet argue that properly collected honey typically has a low protein content [59,60]. Conversely, Somerville suggests that the variation in protein content may be influenced by the type of hive, the extraction method, and the pollen composition of the honey [61].

The analysis of the glucose and protein contents reveals notable disparities. The glucose, for example, varies from 24.11% in carob honeys to 30.32% in rosemary honeys, with intermediate values for jujube and multifloral honeys (27.18% and 29.27% respectively). As for the proteins, they range from 0.173 g/100 g for rosemary honeys to 0.443 g/100 g for jujube honeys. The variability in protein content can be influenced by the type of hive, the extraction method, and the pollen composition of the honey, as suggested by Somerville and other researchers. These analyses highlight the complexity and richness of the chemical profiles of honeys, reflecting their botanical origins and the specific conditions of production.

### 3.3. Antioxidant Activity of Honey

#### 3.3.1. DPPH* Free-Radical-Scavenging Test

**Figure 3:**
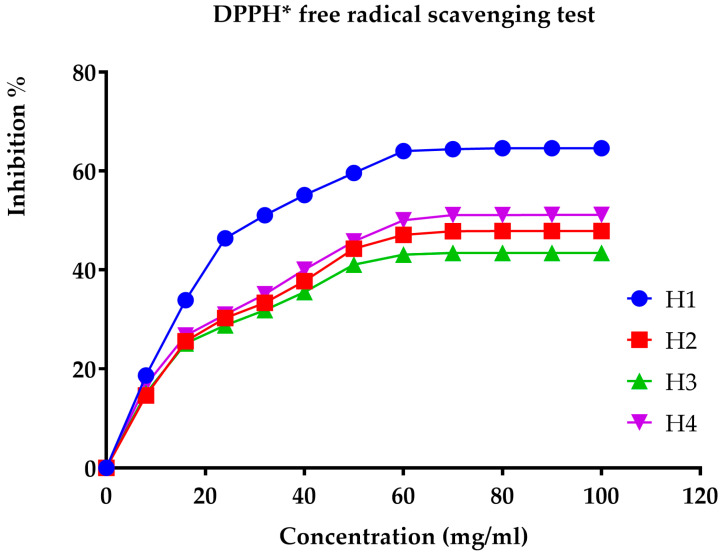
Percentage of inhibition as a function of concentration for multi-flower (**H_1_**), jujube (**H_2_**), carob (**H_3_**), and rosemary (**H_4_**) honeys, assessed using the DPPH* free-radical-scavenging method.

**Figure 4:**
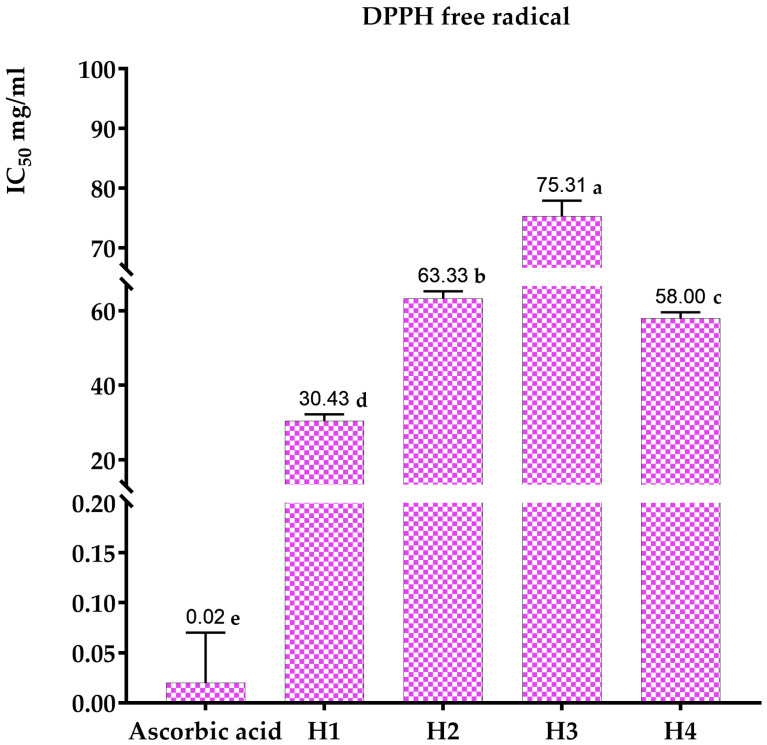
IC_50_ values for ascorbic acid and several types of honey, including multi-flower honey, jujube honey, carob honey, and rosemary honey, to determine their inhibitory effects on DPPH free radicals. The results with different letters are significantly different from each other (*p* < 0.05).

The antioxidant activity of the honey under investigation was assessed utilizing ascorbic acid as the reference standard. The results obtained from the DPPH* assay, which evaluates free-radical-scavenging activity, are presented in Figure 3 and Figure 4. Specifically, Figure 4 consolidates the data on the four types of honey, along with the IC_50_ values for ascorbic acid, representing the concentration required to inhibit 50% of free radicals.

The inhibitory concentration of ascorbic acid is approximately 20 µg/mL. The honey samples’ inhibitory concentrations (IC_50_) are displayed in Figure 4. According to Kanoun [62], multi-flower honey has the strongest antioxidant activity due to its lowest IC_50_ of 30.43 mg/mL. The antioxidant capacities of carob, jujube, and rosemary honey are lower, with IC_50_ values of 75.31 mg/mL, 63.33 mg/mL, and 58 mg/mL, respectively, for Ouazzane honeys (Morocco). Turkish honey has been shown to have inhibitory concentrations ranging from 20.05 mg/mL to 152.40 mg/mL, according to Can [63].

#### 3.3.2. Evaluation of Antioxidant Activity by the FRAP Method

**Figure 5:**
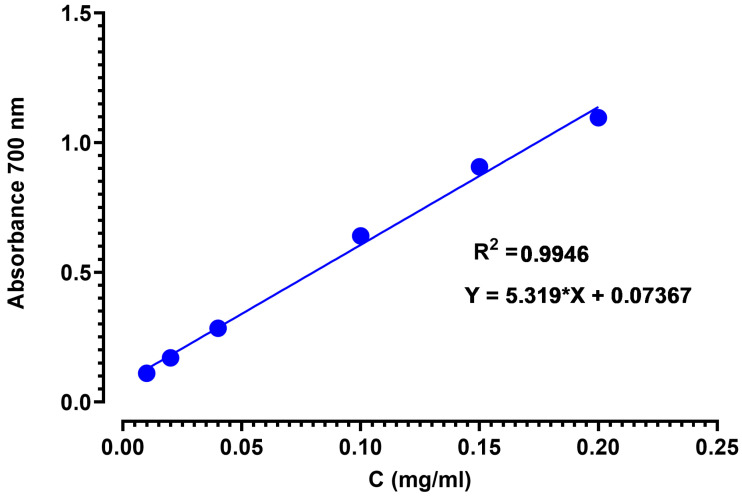
Calibration curve for ascorbic acid using the FRAP method.

Ascorbic acid is the standard antioxidant used to assess the ability of honey to reduce ferric iron Fe^3+^ to ferrous iron Fe^2+^. The calibration curve is shown in Figure 5.

**Figure 6:**
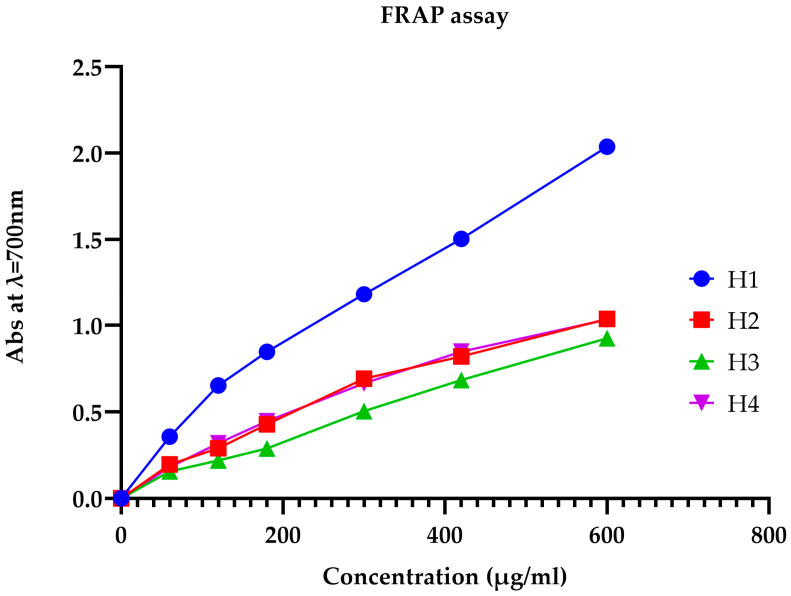
The absorbance of multi-flower honey, jujube honey, carob honey, and rosemary honey as a function of concentration using the FRAP method.

**Figure 7:**
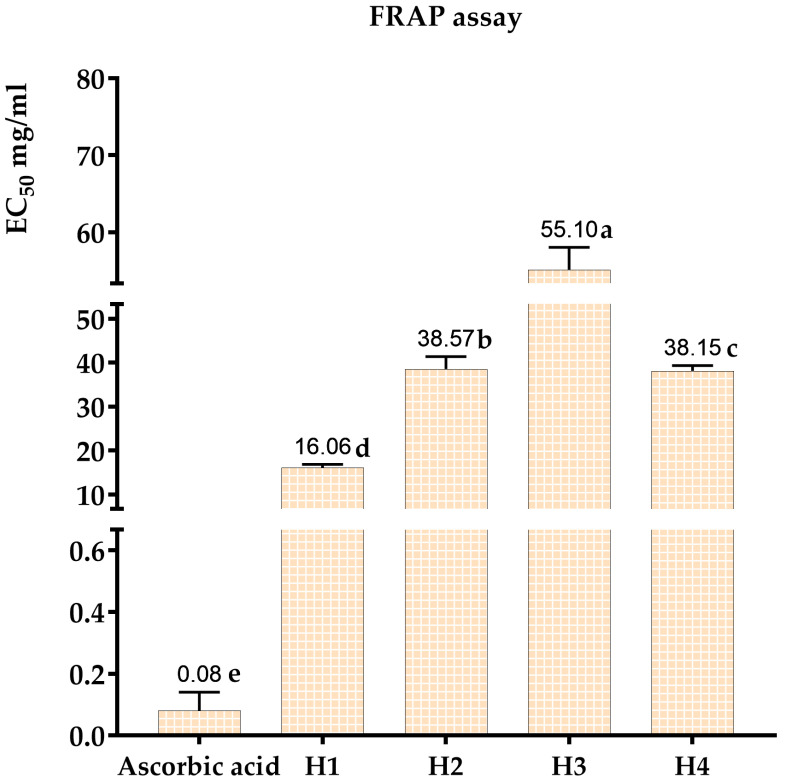
EC_50_ of ascorbic acid and multi-flower honey, jujube honey, carob honey, and rosemary honey (FRAP method). The results with different letters are significantly different from each other (*p* < 0.05).

The most powerful antioxidant is ascorbic acid, which has an effective concentration (EC_50_) of around 0.08 mg/mL. Of all the honeys evaluated, multi-flower honey had the highest antioxidant activity (EC_50_ = 16.06 mg/mL). This was followed, in order of antioxidant activity, by carob honey (EC_50_ = 55.1 mg/mL), jujube honey (EC_50_ = 38.57 mg/mL), and rosemary honey (EC_50_ = 38.15 mg/mL) (Figure 6 and Figure 7). These results are consistent with those obtained using the DPPH* free-radical-trapping technique.

### 3.4. Study of Antimicrobial Activity

The comprehensive antimicrobial activities of the honey varieties are presented in full detail in Table 4. This table provides a clear and concise summary of the minimum inhibitory concentrations (MICs) and minimum bactericidal/fungicidal concentrations (MBCs/MFCs) for each honey sample against the tested bacterial and fungal strains. To provide a comparative reference, the MIC values of the standard antibiotics and the antifungal agent terbinafine are also included in Table 5. This allows for a direct comparison of the antimicrobial potency of the honey samples to that of conventional pharmaceutical agents commonly used to manage microbial infections.

This study revealed antibacterial and antifungal activity in all varieties of honey. Specifically, multi-flower honey proved the most potent against *Enterobacter cloacae*, with a minimum inhibitory concentration (MIC) and minimum bactericidal concentration (MBC) of 6.25 mg/mL. Jujube honey came next, with a MIC and MBC of 12.5 mg/mL. Carob and rosemary honey showed inhibitory concentrations of around 25 mg/mL. The same concentrations were observed for *Klebsiella pneumoniae*, *Escherichia coli*, *Staphylococcus aureus*, and *Staphylococcus epidermidis*. This finding is consistent with previous studies that have reported the broad-spectrum antimicrobial activity of multi-flower honey [64,65,66].

The antifungal properties of multi-flower honey are also noteworthy, as it displayed the highest potency against *Candida albicans*, with an MIC and MBC of 3.125 mg/mL. Candida albicans is a common opportunistic fungal pathogen that can cause serious infections, particularly in immunocompromised individuals [67,68]. The ability of multi-flower honey to effectively inhibit and kill this fungal strain suggests it may have potential as a natural antifungal agent, which could be further explored for therapeutic applications.

Furthermore, the multi-flower honey exhibited strong inhibitory effects against other medically relevant *Candida* species, such as *C. tropicalis* and *C. parapsilosis*, as well as the filamentous fungus *Aspergillus niger*. This finding indicates that multi-flower honey possesses broad-spectrum antifungal activity. Previous studies have reported similar broad-spectrum antifungal properties of honey against various *Candida* and *Aspergillus* species [69,70]. Henriques et al. [71] investigated the antifungal activity of different honeys and found that they were effective in inhibiting the growth of *Candidatropicalis* and *Candida parapsilosis*, in addition to *Candida albicans*. Additionally, Sherlock et al. [72] demonstrated the potent antifungal effects of Ulmo honey from Chile and Manuka honey against *Aspergillus niger*, highlighting the versatility of honey as a natural antifungal agent. The ability of multi-flower honey to inhibit the growth of these diverse fungal pathogens suggests that it may have potential applications in the management of various fungal infections, particularly those caused by opportunistic *Candida* and *Aspergillus* species, which can lead to serious complications in immunocompromised individuals.

In comparison, the other honey varieties (jujube, carob, and rosemary) showed relatively lower antimicrobial potency, with higher MIC and MBC values. However, these honey types still demonstrated significant antibacterial and antifungal activities, suggesting they may also have potential applications as natural antimicrobial agents.

The findings of this study highlight the remarkable antimicrobial properties of honey, particularly multi-flower honey, which demonstrated the most potent antibacterial and antifungal activities. These results warrant further investigation into the potential use of honey, and specifically multi-flower honey, as a natural alternative to conventional antimicrobial agents in various clinical and pharmaceutical applications.

### 3.5. Pearson Correlation between Antioxidant Activity, Antimicrobial Activity, and the Polyphenol and Flavonoid Composition of Four Varieties of Honey

**Figure 8:**
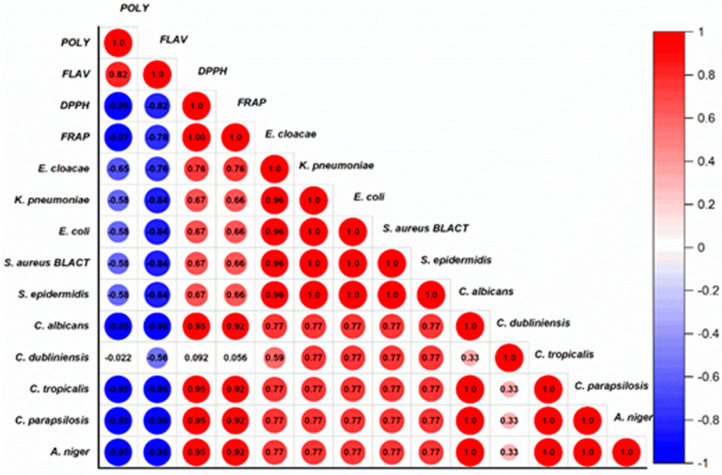
Correlation between the antioxidant activity, antimicrobial activity, and polyphenol and flavonoid composition of four varieties of honey (Poly: polyphenols; Flav: flavonoids).

This comprehensive study provides valuable insights into the relationships between the antioxidant activity, antimicrobial activity, and phytochemical composition of four honey varieties. The use of the Pearson correlation coefficient allows for a robust statistical analysis of these complex interrelationships. Figure 8 displays the findings of the correlation study, with the Bravais–Pearson correlation coefficient (R) ranging from −1 to +1, where a stronger correlation is indicated by a number nearer −1 or +1.

The findings clearly demonstrate a strong positive correlation (R^2^ = 0.82) between the polyphenol and flavonoid contents of the honey samples (Figure 8). This is consistent with previous studies reporting the co-occurrence of these two classes of phytochemicals in honey, as they are often derived from the same botanical sources [73,74,75].

Moreover, the study reveals a strong negative association between polyphenol content and the honeys’ IC_50_ (DPPH) and EC_50_ (FRAP) antioxidant activity concentrations (R^2^ = −0.97 and R^2^ = −0.99, respectively). This discovery supports the well-known function of polyphenols as strong antioxidants in honey, where higher polyphenol concentrations are associated with increased ability to scavenge and reduce free radicals [76,77].

Interestingly, the antifungal efficacy of the polyphenol content was also shown to have a very high negative correlation (R^2^ = −0.95) with the growth of several *Aspergillus* and *Candida* species, except for *Candida dubliniensis* (R^2^ = −0.022). Consistent with earlier results on the antifungal activities of honey phytochemicals, this implies that the polyphenols in honey may be crucial in inhibiting these therapeutically significant fungal infections [1,78,79].

In contrast, the relationship between polyphenol content and antibacterial activity was less straightforward, with only a moderate negative correlation (R^2^ ranging from −0.65 to −0.53) observed for *Enterobacter cloacae*, *Klebsiella pneumoniae*, *Escherichia coli*, *Staphylococcus aureus*, and *Staphylococcus epidermidis*. The discrepancy could be explained by the complex mechanisms of honey’s antibacterial action, which include physicochemical characteristics, enzymatic factors, and other phytochemicals in addition to polyphenols [80,81,82].

Regarding the flavonoid content, a strong inverse correlation was observed with the antioxidant activity of FRAP (R^2^ = −0.78) and DPPH (R^2^ = −0.82). This implies that the role of flavonoids in honey may be more complex and that, in contrast to polyphenols, they may not be the main contributors to total antioxidant capacity. Similar findings from earlier research suggest that honey’s flavonoid content is not the only factor influencing its antioxidant efficacy [80,83,84]. For instance, Beretta et al. [85] found that the antioxidant properties of honey could not be attributed solely to its flavonoid composition, but rather involved a synergistic interplay between various phytochemicals, including polyphenols and other unidentified compounds. Furthermore, Gheldof et al. [86] demonstrated that the antioxidant activity of honey was more strongly correlated with its total phenolic content than its individual flavonoid compounds, suggesting a more complex mechanism of action. These results underline the need for a more thorough comprehension of the distinct functions and interactions of various phytochemicals in adding to honey’s total antioxidant potential.

However, a significant negative correlation (R^2^ = −0.96) was found between flavonoid concentration and antifungal potency, except for *Candida dubliniensis* (R^2^ = −0.56), indicating an important role of flavonoids in honey’s antifungal properties.

Lastly, the study found strong positive correlations between the antioxidant activity (DPPH and FRAP) and the antifungal (R^2^ = 0.95 and 0.92, respectively) and antibacterial (R^2^ = 0.76 for Enterobacter cloacae and R^2^ = 0.66 for other bacterial strains) activities of the honey samples. 

The aforementioned implies that the antibacterial characteristics of honey could be intimately associated with its antioxidant potential, presumably through the combined influence of its phytochemical components. Similar results from earlier research have been published, emphasizing the complex link between honey’s antibacterial and antioxidant properties [87,88,89]. For instance, Alvarez-Suarez et al. [90] found that the antimicrobial capacity of several mono-floral Cuban honeys was strongly correlated with their antioxidant properties, which they attributed to the presence of polyphenols and other phytochemicals. Furthermore, Boukraa [91] reviewed the antimicrobial mechanisms of honey, emphasizing the role of its antioxidant components, such as polyphenols and flavonoids, in inhibiting the growth and virulence of various pathogenic microorganisms. These findings provide a possible explanation for the correlations seen in the current investigation and indicate that the synergistic interaction between the antioxidant and phytochemical contents of honey is important for its overall antibacterial activity.

This study offers convincing proof that honey’s polyphenol and flavonoid contents are closely linked to its antibacterial and antioxidant properties. The possible use of honey as a natural source of antioxidants and antibacterial agents in a variety of applications, such as food preservation, wound healing, and pharmaceutical development, is significantly affected by these discoveries.

### 3.6. Heat Map with Clusters of the Honeys Studied According to Their Polyphenol and Flavonoid Composition and Their Biological Activities

**Figure 9:**
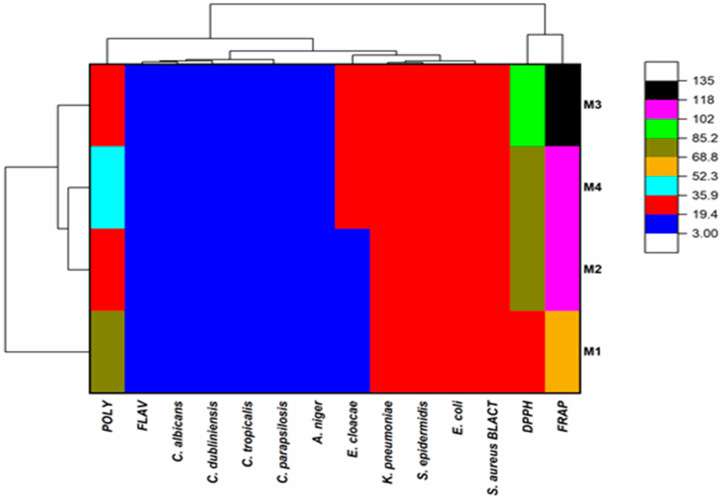
Heat map with clusters of honeys studied as functions of antioxidant activity, antimicrobial activity, and polyphenol and flavonoid composition.

The heat map provides a visually compelling means of interpreting the complex relationships between the phytochemical composition, antioxidant activity, and antimicrobial properties of the honey samples. The use of a color-coded scale, where low frequencies are indicated by red and blue, intermediate frequencies by dark yellow, and high frequencies by black and pink, allows for an intuitive understanding of the data patterns (Figure 9).

The hierarchical clustering displayed in the dendrograms on the left-hand side of the heat map can be used to identify three distinct groups of honey based on their phytochemical and functional characteristics. The first group encompasses the multi-flower honey, the second group includes the jujube and rosemary honeys, and the third group is represented by the carob honey. This clustering aligns with previous reports on the significant variability in the phytochemical profiles and bioactivities of different monofloral and multifloral honey varieties [90,92].

The dendrogram at the top of the heat map can be further divided into three subgroups, providing insights into the specific relationships between the honey samples. The first subgroup is related to the concentration of polyphenols, a class of phytochemicals widely recognized for their potent antioxidant and antimicrobial properties in honey [93,94,95]. 

The second subgroup in the dendrogram at the top of the heat map is associated with the combination of flavonoid content and the honey’s antibacterial and antifungal activities. This finding corroborates the important role of flavonoids in the antimicrobial efficacy of honey. Previous studies have demonstrated the potent antibacterial and antifungal properties of honey flavonoids against a wide range of pathogenic microorganisms [96,97]. For instance, Sherlock et al. [72] reported the significant antimicrobial activity of Ulmo honey from Chile and Manuka honey against methicillin-resistant *Staphylococcus aureus*, *Escherichia coli*, and *Pseudomonas aeruginosa*, which they attributed to the synergistic effects of various phytochemicals, including flavonoids. Similarly, Moussa et al. [67] found that the antifungal activity of different honey types from Algeria was closely linked to their flavonoid content, particularly against the opportunistic fungal pathogens *Candida albicans* and *Rhodotorula species*. These studies provide a solid foundation for understanding the mechanistic contribution of flavonoids to the antimicrobial properties of honey, as further corroborated by the findings of the current heat map analysis. The third subgroup focuses on the antioxidant activity as determined by the FRAP and DPPH assays, which are commonly used to evaluate the free-radical-scavenging and reducing power of honey.

The individual honey samples exhibit distinct patterns within this framework. Honey H_1_ is characterized by a high polyphenol content, which is strongly correlated with its antioxidant activity as measured by the FRAP method. Honey H_2_ displays a substantial correlation with antioxidant activity according to the FRAP technique, but a weaker association based on the DPPH assay, suggesting potential differences in the honey’s reducing and free-radical-scavenging capabilities. In contrast, honey H_4_ has been linked to significant antioxidant activity, as shown by both the FRAP and DPPH techniques, likely due to its higher polyphenol content. The greatest correlation between the FRAP and DPPH methods was observed for honey H_3_, further emphasizing the complexity and variability in the antioxidant profiles of different honey samples.

The heat map analysis provides a comprehensive visual representation of the intricate relationships between the phytochemical composition, antioxidant activity, and antimicrobial properties of the honey varieties investigated. These findings contribute to a deeper understanding of the factors that contribute to the functional attributes of honey, with potential implications for the development of natural antimicrobial and antioxidant agents derived from this valuable natural resource.

## 4. Conclusions

The results of this study offer crucial knowledge to the honey sector in Morocco. We have conducted a thorough analysis of the physical and chemical characteristics, as well as the chemical compounds, antioxidant activity, and ability to inhibit the growth of microorganisms, of four types of honey (multifloral, carob, rosemary, jujube) from the Fez-Meknes area.

While the majority of the honey samples conform to the requirements established by the Codex Alimentarius, we have seen notable variations that may be attributed to the specific plant species and geographical locations from which each type originates. The distinct attributes of various honeys, such as pH, free acidity, density, and HMF concentration, contribute to their individual aromatic profiles and functional properties.

The examination of phytochemicals showed that multifloral honey has the greatest levels of flavonoids and polyphenols, which contribute to its exceptional antioxidant and antibacterial characteristics. Jujube, carob, and rosemary honeys are notable for their abundance of bioactive components, which contribute to their strong antioxidant and antibacterial properties.

An important innovation of this study is the discovery of significant correlations between the levels of polyphenols/flavonoids and the biological effects of honey. The findings indicate that the antioxidant and antibacterial characteristics of honey can be linked to its phytochemical content. This discovery presents opportunities for utilizing honey as a natural substitute for synthetic substances in the cosmetic and medicinal fields.

Further investigations should focus on undertaking more detailed chromatographic examinations of the different phenolic compounds found in these honeys in order to gain a more comprehensive understanding of their unique roles in the reported activities. Furthermore, investigating variations in honey content over time in response to environmental shifts and beekeeping methods would be a relevant field of study.

## Figures and Tables

**Table 1:** Origin and provenance of honey samples.

Sample Coding	Botanical Origin	Localization	Coordinates	Harvest Date
**H_1_**	Multi flower	Zaouia Ifrane	33°31′60″ North 5°7′0″ West	July 2021
**H_2_**	Jujube	Bouderbala Meknes	33°49′0″ North 5°16′33″ West	July 2021
**H_3_**	Carob	Moulay Driss Zerhoun	34°3′15.00″ North 5°31′38.00″ West	July 2021
**H_4_**	Rosemary	Oulad Ali Youssef Boulemane	33°23′43″ North 3°34′53″ West	July 2021

**Table 2:** Bacterial and fungal strains tested.

Bacterial Strains	References	Fungal Strains	References
*Enterobacter cloacae*	02EV317	*Candida albicans*	Ca
*Klebsiella pneumoniae*	3DT1823	*Candida dubliniensis*	Cd
*Escherichia coli sauvage*	3DT1938	*Candida tropicalis*	Ct
*Staphylococcus aureus BLACT*	4IH2510	*Candida parapsilosis*	Cpa
*Staphylococcus epidermidis*	5994	*Aspergillus niger*	AspN

**Table 3:** Physicochemical characteristics of multi-flower honey (H_1_), jujube honey (H_2_), carob honey (H_3_), and rosemary honey (H_4_).

Ref.	pH	Free Acidity (meq/kg)	Brix Degree (%)	Water Content (%)	Density at 20 °C (g/mL)	Ash Content (%)	Conductivity (mS/cm)	Hydroxymethylfurfurale HMF (mg/Kg)	Color Intensity, ABS450 (mAU, 50 *w*/*v*)
H_1_	3.34 ± 0.02	13 ± 0.10	80.2 ± 0.38	19.8 ± 0.38	1.412 ± 0.01	0.29 ± 0.01	0.199 ± 0.01	37.5 ± 0.24	1202 ± 8
H_2_	3.09 ± 0.01	11 ± 0.30	81.2 ± 0.46	18.8 ± 0.31	1.421 ± 0.01	0.6 ± 0.01	0.473 ± 0.01	35.5 ± 0.26	622 ± 6
H_3_	3.51 ± 0.02	8 ± 0.10	82.1 ± 0.41	17.9 ± 0.42	1.437 ± 0.01	0.59 ± 0.01	0.372 ± 0.01	30.34 ± 0.44	558 ± 4
H_4_	2.91 ± 0.01	9 ± 0.20	81.2 ± 0.31	18.8 ± 0.36	1.426 ± 0.01	0.31 ± 0.01	0.256 ± 0.01	48.65 ± 0.51	313 ± 3
Codex Alimentarius standards 2001siem [42]	Nectar honey: [3.5–4.5]. Honeydew honey: [5–5.5]	˂50	-	≤20	[1.39–1.44]	Nectar honey: ≤0.6	Nectar honey: ≤0.8. Honeydew honey: >0.8	˂40	-

**Table 4:** MICs of antibiotics and antifungals (µg/mL) against the microbial strains studied.

Strains		*Antibiotics* *				*Antifungals * ^#^
		*Gentamycin*	*Amoxicillin–Clavulanate*	*Vancomycin*	*Trimethoprim-Sulfamethoxazole*	*Terbinafine*
***Bacteria***	***S.*** ***epidermidis***	2		>8	>4/76	
	***S. aureus BLACT***	<0.5		2	<10	
	***E.*** ***coli***	2	8/2		≤1/19	
	***E. cloacae***	>4	>8/2		>4/76	
	***K.*** ***pneumoniae***	≤1	≤2/2		≤1/19	
***Yeasts***	***C.*** ***albicans***					12.500
	***C.*** ***parapsilosis***					6.250
	***C.*** ***tropicalis***					12.500
	***C.*** ***dubliniensis***					3.125
***Molds***	***A.*** ***niger***					3.125

*: the MIC (µg/mL) of the antibiotics was determined by the BD Phoenix™ identification and antibiogram instrument; ^#^: the MIC (µg/mL) of terbinafine was determined on a microplate.

**Table 5:** MICs (mg/mL) and MBCs (mg/mL) of four varieties of honey: multi-flower honey (H_1_), jujube honey (H_2_), carob honey (H_3_) and rosemary honey (H_4_).

Strains		H_1_		H_2_		H_3_		H_4_	
		MIC	MBC	MIC	MBC	MIC	MBC	MIC	MBC
**Bacteria**	*Enterobactercloacae*	6.25	6.25	12.5	12.5	25	25	25	25
	*Klebsiellapneumoniae*	25	25	25	25	25	25	25	25
	*Escherichia coli sauvage*	25	25	25	25	25	25	25	25
	*Staphylococcus aureus BLACT*	25	25	25	25	25	25	25	25
	*Staphylococcus epidermidis*	25	25	25	25	25	25	25	25
**Fungi**	*Candida albicans*	3.125	3.125	6.25	6.25	6.25	6.25	6.25	6.25
	*Candida dubliniensis*	6.25	6.25	6.25	6.25	6.25	6.25	12.5	12.5
	*Candida tropicalis*	6.25	6.25	12.5	12.5	12.5	12.5	12.5	12.5
	*Candida parapsilosis*	6.25	6.25	12.5	12.5	12.5	12.5	12.5	12.5
	*Aspergillus niger*	6.25	6.25	12.5	12.5	12.5	12.5	12.5	12.5

## Data Availability

Data are contained within the article.

## References

[1] Cianciosi D., Forbes-Hernández T.Y., Afrin S., Gasparrini M., Reboredo-Rodriguez P., Manna P.P., Zhang J., Bravo Lamas L., Martínez Flórez S., Agudo Toyos P. (2018). Phenolic Compounds in Honey and Their Associated Health Benefits: A Review. Molecules.

[2] Uttara B., Singh A.V., Zamboni P., Mahajan R.T. (2009). Oxidative Stress and Neurodegenerative Diseases: A Review of Upstream and Downstream Antioxidant Therapeutic Options. Curr. Neuropharmacol..

[3] Scalbert A., Manach C., Morand C., Rémésy C., Jiménez L. (2005). Dietary Polyphenols and the Prevention of Diseases. Crit. Rev. Food Sci. Nutr..

[4] Han X., Shen T., Lou H. (2007). Dietary Polyphenols and Their Biological Significance. Int. J. Mol. Sci..

[5] Bueno-Costa F.M., Zambiazi R.C., Bohmer B.W., Chaves F.C., da Silva W.P., Zanusso J.T., Dutra I. (2016). Antibacterial and Antioxidant Activity of Honeys from the State of Rio Grande Do Sul, Brazil. LWT Food Sci. Technol..

[6] Gül A., Pehlivan T. (2018). Antioxidant Activities of Some Monofloral Honey Types Produced across Turkey. Saudi J. Biol. Sci..

[7] Olas B. (2020). Honey and Its Phenolic Compounds as an Effective Natural Medicine for Cardiovascular Diseases in Humans?. Nutrients.

[8] Doukani K., Souhila T., Asma D., Zahira H. (2014). Etude Physicochimique et Phytochimique de Quelques Types de Miels Algériens. Rev. Ecol.-Environ..

[9] Balas F. Les propriétés thérapeutiques du miel et leurs domaines d’application en médecine générale: Revue de la littérature. 2015, 85. https://core.ac.uk/download/pdf/52773796.pdf.

[10] Imtara H., Elamine Y., Lyoussi B. (2018). Honey Antibacterial Effect Boosting Using *Origanum Vulgare* L. Essential Oil. Evid.-Based Complement. Altern. Med. ECAM.

[11] Kaznowski A., Szymas B., Jazdzinska E., Kazimierczak M., Paetz H., Mokracka J. (2005). The Effects of Probiotic Supplementation on the Content of Intestinal Microflora and Chemical Composition of Worker Honey Bees (*Apis mellifera*). J. Apic. Res..

[12] Saranraj P., Sivasakthi S. (2018). Comprehensive Review on Honey: Biochemical and Medicinal Properties. J. Acad. Ind. Res..

[13] Gholizadeh H., Ghaffarifar F., Dalimi A., Dayer M.S. (2022). In Vitro and in Vivo Effects of Natural Honey on Leishmania Major. Ann. Parasitol..

[14] Zeina B., Othman O., al-Assad S. (1996). Effect of Honey versus Thyme on Rubella Virus Survival in Vitro. J. Altern. Complement. Med..

[15] Ghapanchi J., Moattari A., Andisheh Tadbir A., Talatof Z., Shahidi S.P., Ebrahimi H. (2011). The In Vitro Anti-Viral Activity of Honey on Type 1 Herpes Simplex Virus. Aust. J. Basic Appl. Sci..

[16] Grabek-Lejko D., Miłek M., Sidor E., Puchalski C., Dżugan M. (2022). Antiviral and Antibacterial Effect of Honey Enriched with *Rubus* Spp. as a Functional Food with Enhanced Antioxidant Properties. Molecules.

[17] Almeida-Muradian L., Barth O., Dietemann V., Eyer M., Freitas A., Martel A.-C., Marcazzan G., Marchese C., Mucignat-Caretta C., Pascual Maté A. (2020). Standard Methods for Apis Mellifera Honey Research. J. Apic. Res..

[18] Donkersley P., Rhodes G., Pickup R.W., Jones K.C., Power E.F., Wright G.A., Wilson K. (2017). Nutritional Composition of Honey Bee Food Stores Vary with Floral Composition. Oecologia.

[19] da Silva P.M., Gauche C., Gonzaga L.V., Costa A.C.O., Fett R. (2016). Honey: Chemical Composition, Stability and Authenticity. Food Chem..

[20] Valverde S., Ares A.M., Stephen Elmore J., Bernal J. (2022). Recent Trends in the Analysis of Honey Constituents. Food Chem..

[21] Liu T., Ming K., Wang W., Qiao N., Qiu S., Yi S., Huang X., Luo L. (2021). Discrimination of Honey and Syrup-Based Adulteration by Mineral Element Chemometrics Profiling. Food Chem..

[22] Rossant A. (2011). Le Miel: Un Composé Complexe Aux Propriétés Surprenantes = Honey, a Complex Compound with Surprising Properties. Ph.D. Thesis.

[23] De-Melo A., Almeida-Muradian L., Sancho M., Pascual Maté A. (2017). Composition and Properties of Apis Mellifera Honey: A Review. J. Apic. Res..

[24] Chirife J., Zamora M.C., Motto A. (2006). The Correlation between Water Activity and % Moisture in Honey: Fundamental Aspects and Application to Argentine Honeys. J. Food Eng..

[25] Mijanur Rahman M., Gan S.H., Khalil M.d.I. (2014). Neurological Effects of Honey: Current and Future Prospects. Evid.-Based Complement. Altern. Med. ECAM.

[26] El-Sohaimy S., Masry S., Shehata M. (2015). Physicochemical Characteristics of Honey from Different Origins. Ann. Agric. Sci..

[27] Bogdanov S., Martin P., Lüllmann C. (1997). Harmonised Methods of the European Honey Com-Mission. Apidologie Extra.

[28] Anklam E. (1998). A Review of the Analytical Methods to Determine the Geographical and Botanical Origin of Honey. Food Chem..

[29] Bouhlali E.D.T., Bammou M., Sellam K., El Midaoui A., Bourkhis B., Ennassir J., Alem C., Filali-Zegzouti Y. (2019). Physicochemical Properties of Eleven Monofloral Honey Samples Produced in Morocco. Arab J. Basic Appl. Sci..

[30] White J.W., Chichester C.O. (1978). Honey. Advances in Food Research.

[31] Kocyigit A., Aydogdu G., Balkan E., Yenigun V.B., Guler E.M., Bulut H., Koktasoglu F., Gören A.C., Atayoglu A.T. (2019). Quercus Pyrenaica Honeydew Honey With High Phenolic Contents Cause DNA Damage, Apoptosis, and Cell Death Through Generation of Reactive Oxygen Species in Gastric Adenocarcinoma Cells. Integr. Cancer Ther..

[32] Bradford M.M. (1976). A Rapid and Sensitive Method for the Quantitation of Microgram Quantities of Protein Utilizing the Principle of Protein-Dye Binding. Anal. Biochem..

[33] Singleton V.L., Orthofer R., Lamuela-Raventós R.M. (1999). [14] Analysis of Total Phenols and Other Oxidation Substrates and Antioxidants by Means of Folin-Ciocalteu Reagent. Methods in Enzymology.

[34] Hung P.V., Maeda T., Miyatake K., Morita N. (2009). Total Phenolic Compounds and Antioxidant Capacity of Wheat Graded Flours by Polishing Method. Food Res. Int..

[35] Djeridane A., Yousfi M., Nadjemi B., Boutassouna D., Stocker P., Vidal N. (2006). Antioxidant Activity of Some Algerian Medicinal Plants Extracts Containing Phenolic Compounds. Food Chem..

[36] Brand-Williams W., Cuvelier M.E., Berset C. (1995). Use of a Free Radical Method to Evaluate Antioxidant Activity. LWT Food Sci. Technol..

[37] Zovko Končić M., Kremer D., Karlović K., Kosalec I. (2010). Evaluation of Antioxidant Activities and Phenolic Content of *Berberis Vulgaris* L. and *Berberis Croatica* Horvat. Food Chem. Toxicol..

[38] Balouiri M., Sadiki M., Ibnsouda S.K. (2016). Methods for in Vitro Evaluating Antimicrobial Activity: A Review. J. Pharm. Anal..

[39] Julika W.N., Ajit A., Sulaiman A.Z., Naila A. (2019). Physicochemical and Microbiological Analysis of Stingless Bees Honey Collected from Local Market in Malaysia. Indones. J. Chem..

[40] EUCAST EUCAST (2023). Breakpoint Tables for Interpretation of MICs and Zone Diameters, Ver. 13.0.

[41] (2017). EUCAST Guidelines for Detection of Resistance Mechanisms and Specific Resistances of Clinical and/or Epidemiological Importance, Ver. 2.0.

[42] Food and Agriculture Organization of the United Nations, World Health Organization Joint FAO/WHO Food Standard Programme Codex Alimentarius Commission. Proceedings of the Twenty-Fourth Session.

[43] Naman Malika N.M., Faid Mohamed F.M., El-Adlouni Chakib E.-A.C. (2005). Microbiological and Physico-Chemical Properties of Moroccan Honey. Int. J. Agric. Biol..

[44] Iritie B.M., Wandan E.N., Yapo M.Y., Fantodji A., Bodji N.C. (2014). Comparative Analysis of Physico-Chemical Characteristics of Honeys Produced in the Multi-Floral Arboretum of the National School of Agronomy of Yamoussoukro. Int. J. Agric. Policy Res..

[45] Kouamé K.F., Gbouhoury E.K.B., Fofié N.B.Y., Kassi N.J. (2021). Caractéristiques Physicochimiques Récoltés Des Miels De La Sous-Préfecture De Cechi (Dans Le Département D’agboville, Côte D’ivoire). Eur. Sci. J. ESJ.

[46] Bakchiche B., Habati M., Benmebarek A., Gherib A. (2018). Caractéristiques Physico-Chimiques, Concentrations des Composés Phénoliques et Pouvoir Antioxydant de Quatre Variétés de Miels Locales (Algérie). Rev. Marocaine Sci. Agron. Vét..

[47] Chen C. (2019). Relationship between Water Activity and Moisture Content in Floral Honey. Foods.

[48] Ouchemoukh S., Louaileche H., Schweitzer P. (2007). Physicochemical Characteristics and Pollen Spectrum of Some Algerian Honeys. Food Control.

[49] Jean-Prost P. (1987). Apiculture; Connaitre l’abeille, Conduire Le Rucher.

[50] Fechner D.C., Moresi A.L., Díaz J.D.R., Pellerano R.G., Vazquez F.A. (2016). Multivariate Classification of Honeys from Corrientes (Argentina) According to Geographical Origin Based on Physicochemical Properties. Food Biosci..

[51] Imtara H., Elamine Y., Lyoussi B. (2018). Physicochemical Characterization and Antioxidant Activity of Palestinian Honey Samples. Food Sci. Nutr..

[52] Al-Mamary M.A. (2002). Antioxidant Activity of Commonly Consumed Vegetables in Yemen. Malays. J. Nutr..

[53] Perna A., Simonetti A., Intaglietta I., Sofo A., Gambacorta E. (2012). Metal Content of Southern Italy Honey of Different Botanical Origins and Its Correlation with Polyphenol Content and Antioxidant Activity. Int. J. Food Sci. Technol..

[54] Bouyahya A., Dakka N., Talbaoui A., Moussaoui N.E., Abrini J., Bakri Y. (2018). Phenolic Contents and Antiradical Capacity of Vegetable Oil from *Pistacia lentiscus* (L). J. Mater. Environ. Sci..

[55] Šarić G., Marković K., Major N., Krpan M., Uršulin-Trstenjak N., Hruškar M., Vahčić N. (2012). Changes of Antioxidant Activity and Phenolic Content in Acacia and Multifloral Honey During Storage. Food Technol. Biotechnol..

[56] Kadri S.M., Zaluski R., Lima G.P.P., Mazzafera P., de Oliveira Orsi R. (2016). Characterization of Coffea Arabica Monofloral Honey from Espírito Santo, Brazil. Food Chem..

[57] Habib H.M., Al Meqbali F.T., Kamal H., Souka U.D., Ibrahim W.H. (2014). Physicochemical and Biochemical Properties of Honeys from Arid Regions. Food Chem..

[58] Benhamou N., Rey P. (2012). Stimulateurs Des Défenses Naturelles Des Plantes: Une Nouvelle Stratégie Phytosanitaire Dans Un Contexte d’écoproduction Durable. I. Principes de La Résistance Induite. Phytoprotection.

[59] Gonnet M. (1982). Le Miel. Composition Propriétés et Conservation.

[60] White J.W., Riethof M.L., Subers M.H., Kushnir I. (1962). Composition of American Honeys.

[61] Somerville D. (2005). Fat Bees Skinny Bees.

[62] Kanoun K. (2011). Contribution à l’étude Phytochimique et Activité Antioxydante Des Extraits de *Myrtus communis* L. (Rayhane) de La Région de Tlemcen (Honaine). Mém. Magister Univ. Aboubekr Belkaid–Tlemcen.

[63] Can Z., Yildiz O., Sahin H., Turumtay E.A., Silici S., Kolayli S. (2015). An Investigation of Turkish Honeys: Their Physico-Chemical Properties, Antioxidant Capacities and Phenolic Profiles. Food Chem..

[64] Abdellah F. (2023). Antimicrobial Properties of Natural Honey. Melittology-New Advances.

[65] Al-Kafaween M.A., Alwahsh M., Mohd Hilmi A.B., Abulebdah D.H. (2023). Physicochemical Characteristics and Bioactive Compounds of Different Types of Honey and Their Biological and Therapeutic Properties: A Comprehensive Review. Antibiotics.

[66] Halawani E., Shohayeb M. (2011). Survey of the Antibacterial Activity of Saudi and Some International Honeys. J. Microbiol. Antimicrob..

[67] Moussa A., Noureddine D., Saad A., Abdelmelek M., Abdelkader B. (2012). Antifungal Activity of Four Honeys of Different Types from Algeria against Pathogenic Yeast: *Candida albicans* and *Rhodotorula* sp.. Asian Pac. J. Trop. Biomed..

[68] Estevinho M.L., Afonso S.E., Feás X. (2011). Antifungal Effect of Lavender Honey against Candida Albicans, Candida Krusei and Cryptococcus Neoformans. J. Food Sci. Technol..

[69] Candiracci M., Citterio B., Diamantini G., Blasa M., Accorsi A., Piatti E. (2011). Honey Flavonoids, Natural Antifungal Agents Against Candida Albicans. Int. J. Food Prop..

[70] Anyanwu C. (2012). Investigation of in Vitro Antifungal Activity of Honey. J. Med. Plants Res..

[71] Henriques A., Jackson S., Cooper R., Burton N. (2006). Free Radical Production and Quenching in Honeys with Wound Healing Potential. J. Antimicrob. Chemother..

[72] Sherlock O., Dolan A., Athman R., Power A., Gethin G., Cowman S., Humphreys H. (2010). Comparison of the Antimicrobial Activity of Ulmo Honey from Chile and Manuka Honey against Methicillin-Resistant *Staphylococcus aureus*, *Escherichia coli* and *Pseudomonas aeruginosa*. BMC Complement. Altern. Med..

[73] İzol E. (2024). Phytochemicals in Honey and Health Effects. 2023; pp. 85–96. ISBN 9786256598034. https://www.researchgate.net/profile/Ebubekir-Izol/publication/374873469_Phytochemicals_in_Honey_and_Health_Effects/links/653369691d6e8a70703fef7a/Phytochemicals-in-Honey-and-Health-Effects.pdf.

[74] Bernklau E., Bjostad L., Hogeboom A., Carlisle A., Seshadri A. (2019). Dietary Phytochemicals, Honey Bee Longevity and Pathogen Tolerance. Insects.

[75] Zammit Young G.-W., Blundell R. (2023). A Review on the Phytochemical Composition and Health Applications of Honey. Heliyon.

[76] Khalil M.L., Sulaiman S.A. (2010). The Potential Role of Honey and Its Polyphenols in Preventing Heart Disease: A Review. Afr. J. Tradit. Complement. Altern. Med..

[77] Schramm D.D., Karim M., Schrader H.R., Holt R.R., Cardetti M., Keen C.L. (2003). Honey with High Levels of Antioxidants Can Provide Protection to Healthy Human Subjects. J. Agric. Food Chem..

[78] Ayoub W.S., Ritu, Zahoor I., Dar A.H., Farooq S., Mir T.A., Ganaie T.A., Srivastava S., Pandey V.K., Altaf A. (2023). Exploiting the Polyphenolic Potential of Honey in the Prevention of Chronic Diseases. Food Chem. Adv..

[79] Uthurry C., Hevia D., Gomez-Cordoves C. (2011). Role of Honey Polyphenols in Health. J. ApiProduct ApiMedical Sci..

[80] Combarros-Fuertes P., Fresno J.M., Estevinho M.M., Sousa-Pimenta M., Tornadijo M.E., Estevinho L.M. (2020). Honey: Another Alternative in the Fight against Antibiotic-Resistant Bacteria?. Antibiotics.

[81] Almasaudi S. (2021). The Antibacterial Activities of Honey. Saudi J. Biol. Sci..

[82] Cebrero G., Sanhueza O., Pezoa M., Báez M., Martínez J., Báez M., Fuentes E. (2020). Relationship among the Minor Constituents, Antibacterial Activity and Geographical Origin of Honey: A Multifactor Perspective. Food Chem..

[83] Pyrzynska K., Biesaga M. (2009). Analysis of Phenolic Acids and Flavonoids in Honey. TrAC Trends Anal. Chem..

[84] Moniruzzaman M., Yung An C., Rao P.V., Hawlader M.N.I., Azlan S.A.B.M., Sulaiman S.A., Gan S.H. (2014). Identification of Phenolic Acids and Flavonoids in Monofloral Honey from Bangladesh by High Performance Liquid Chromatography: Determination of Antioxidant Capacity. BioMed Res. Int..

[85] Beretta G., Granata P., Ferrero M., Orioli M., Maffei Facino R. (2005). Standardization of Antioxidant Properties of Honey by a Combination of Spectrophotometric/Fluorimetric Assays and Chemometrics. Anal. Chim. Acta.

[86] Gheldof N., Wang X.-H., Engeseth N.J. (2002). Identification and Quantification of Antioxidant Components of Honeys from Various Floral Sources. J. Agric. Food Chem..

[87] Stefanis C., Stavropoulou E., Giorgi E., Voidarou C., Constantinidis T., Vrioni G., Tsakris A. (2023). Honey’s Antioxidant and Antimicrobial Properties: A Bibliometric Study. Antioxidants.

[88] Alvarez-Suarez J.M., Gasparrini M., Forbes-Hernández T.Y., Mazzoni L., Giampieri F. (2014). The Composition and Biological Activity of Honey: A Focus on Manuka Honey. Foods.

[89] Viuda-Martos M., Ruiz-Navajas Y., Fernández-López J., Pérez-Álvarez J.A. (2008). Antibacterial Activity of Different Essential Oils Obtained from Spices Widely Used in Mediterranean Diet. Int. J. Food Sci. Technol..

[90] Alvarez-Suarez J.M., Tulipani S., Díaz D., Estevez Y., Romandini S., Giampieri F., Damiani E., Astolfi P., Bompadre S., Battino M. (2010). Antioxidant and Antimicrobial Capacity of Several Monofloral Cuban Honeys and Their Correlation with Color, Polyphenol Content and Other Chemical Compounds. Food Chem. Toxicol..

[91] Boukraâ L. (2023). Honey in Traditional and Modern Medicine.

[92] Küçük M., Kolaylı S., Karaoğlu Ş., Ulusoy E., Baltacı C., Candan F. (2007). Biological Activities and Chemical Composition of Three Honeys of Different Types from Anatolia. Food Chem..

[93] Al-Mamary M., Al-Meeri A., Al-Habori M. (2002). Antioxidant Activities and Total Phenolics of Different Types of Honey. Nutr. Res..

[94] Patouna A., Vardakas P., Skaperda Z., Spandidos D.A., Kouretas D. (2023). Evaluation of the Antioxidant Potency of Greek Honey from the Taygetos and Pindos Mountains Using a Combination of Cellular and Molecular Methods. Mol. Med. Rep..

[95] Derwich E., Benziane Z., Boukir A. (2009). Chemical Compositions and Insectisidal Activity of Essential Oils of Three Plants *Artemisia* Sp: Artemisia Herba-Alba, Artemisia Absinthium and Artemisia Pontica (Morocco). Electron. J. Environ. Agric. Food Chem..

[96] François E.A., Bertin G., Armand P., Durand M.D.-N., Elvire G., Farid B.-M., Madjid A., Latifou L., Victorien D., Lamine B.-M. (2018). Polyphenolic Profile, and Antioxidant and Antifungal Activities of Honey Products in Benin. Afr. J. Microbiol. Res..

[97] Mohammed S.E.A., El-Niweiri M., AL-Shehri B., Ghramh H., Khan K.A., Elimam M., Kabbashi A., Koko W. (2024). Antimicrobial, Antioxidant and Cytotoxic Properties of Four Types of Honey as Related to Their Phenolic and Flavonoid Contents. Pharmacol. Clin. Pharm. Res..

